# Middle segment-preserving pancreatectomy for metachronous intraductal papillary mucinous neoplasm after pancreatoduodenectomy: a case report

**DOI:** 10.1186/s40792-017-0306-2

**Published:** 2017-02-14

**Authors:** Mihoko Yamada, Teiichi Sugiura, Yukiyasu Okamura, Takaaki Ito, Yusuke Yamamoto, Ryo Ashida, Katsuhiko Uesaka

**Affiliations:** Division of Hepato-Biliary-Pancreatic Surgery, Shizuoka Cancer Center, Shizuoka, Shizuoka Japan

**Keywords:** Middle segment-preserving pancreatectomy, Intraductal papillary mucinous neoplasm

## Abstract

Total pancreatectomy has occasionally been performed to treat patients with multiple lesions (such as intraductal papillary mucinous neoplasm (IPMN)) or patients who have undergone repeated pancreatic resection. However, deficiencies of the exocrine and endocrine functions worsen patients’ quality of life. Recently, there have been several case reports citing middle segment-preserving pancreatectomy (MSPP) as a safe procedure and beneficial with respect to preservation of the exocrine and endocrine functions. We herein report the case of a patient who underwent MSPP for repeat pancreatectomy for IPMN and in whom a favorable outcome was achieved. The patient, a 70-year-old man, was diagnosed with branch duct-type IPMN (BD-IPMN) with worrisome features in the pancreatic head and a single cyst in the pancreatic tail, during a preoperative examination of early gastric cancer. Pancreatoduodenectomy was performed for BD-IPMN in the pancreatic head and gastric cancer. A histopathological examination showed an intraductal papillary mucinous adenoma (IPMA) with mild-moderate atypia. During the follow-up, the size of the cystic lesion in the pancreatic tail and the diameter of the main pancreatic duct were gradually increasing. Therefore, at 2 years and 6 months after surgery, distal pancreatectomy with preservation of the spleen (namely MSPP) was performed. The pancreatic resection margin was histologically negative. The length and volume of the remnant pancreas were approximately 6 cm and 10 ml, respectively. A histopathological examination showed an IPMA. The patient had no diarrhea or weight loss without digestive enzymes and maintained favorable glucose tolerance without oral hypoglycemic agents or insulin. He has showed no evidence of new lesions in the remnant pancreas at 3 years of follow-up after the last surgery.

## Background

Intraductal papillary mucinous neoplasm (IPMN) showed synchronous or metachronous multifocal occurrence in approximately 20% of such patients [1]. The standard treatment protocol is resection for the high risk of malignancy and careful follow-up for the remaining low-risk lesions [2]. In patients with multiple lesions, total pancreatectomy (TP) has occasionally been performed to achieve curative resection. Although the quality of life (QOL) has become acceptable after TP due to improvements in the post-surgery management [3, 4], TP can lead to diabetes mellitus (DM) and related complications [5]. Recently, several case reports have described middle segment-preserving pancreatectomy (MSPP) as a safe procedure providing a favorable outcome with respect to preservation of the exocrine and endocrine functions [6]. We herein report a patient who underwent MSPP for repeat pancreatectomy of IPMN in whom a favorable outcome was achieved.

## Case presentation

**Fig. 1:**
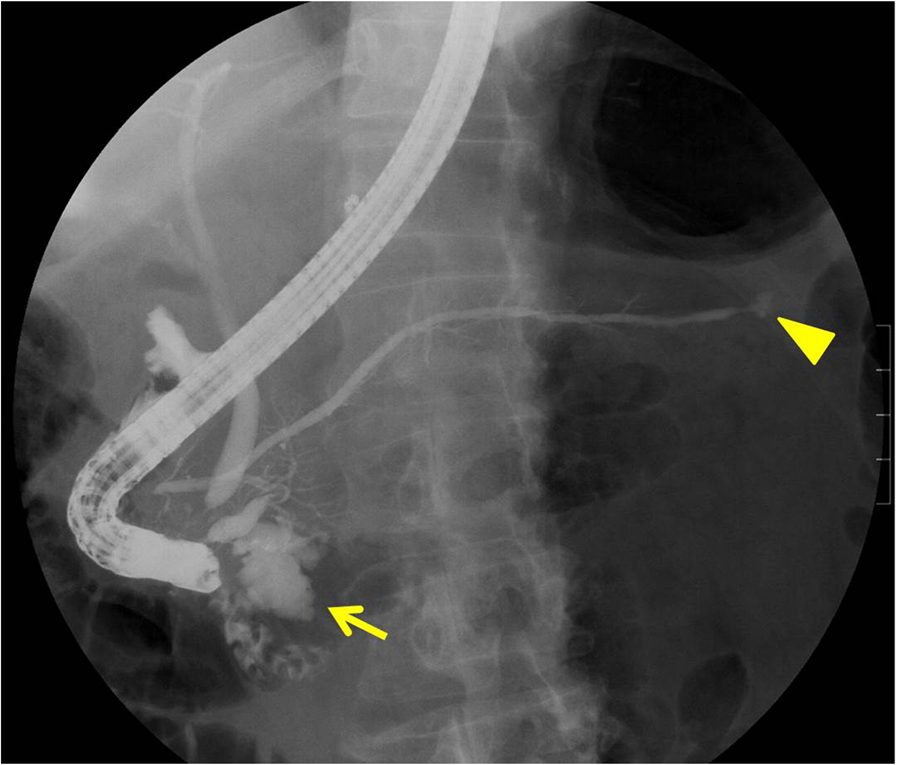
Endoscopic retrograde cholangiopancreatography. There were a 33-mm-diameter multilocular cystic lesion in the pancreatic head (*arrow*) and a 5-mm-diameter monolocular cyst in the pancreatic tail (*head of arrow*). The main pancreatic duct was slightly dilated at 4.5 mm

A 70-year-old man was referred to our hospital for early gastric cancer of the antrum. Abdominal computed tomography (CT) for the preoperative staging showed cystic lesions in the pancreatic head and tail. Therefore, a further examination was performed. Magnetic resonance cholangiopancreatography (MRCP) and endoscopic retrograde cholangiopancreatography (ERCP) showed a 33-mm-diameter multilocular cystic lesion in the pancreatic head and a 5-mm-diameter monolocular cyst in the pancreatic tail (Fig. 1). The main pancreatic duct (MPD) was slightly dilated at 4.5 mm. Endoscopic ultrasonography (EUS) revealed a 7-mm-diameter intramural nodule in the cystic lesion of the pancreatic head and no nodules in that of the pancreatic tail. The lesion in the pancreatic head was diagnosed as a branch duct-type IPMN (BD-IPMN) with worrisome features with a distinct nodule on EUS. It was considered to be an indication for surgery. In contrast, the lesion in the pancreatic tail was judged to be a low-risk lesion with no indication for surgery. Therefore, pancreatoduodenectomy (PD) was performed for the BD-IPMN in the pancreatic head and gastric cancer. The left gastric artery and vein were each ligated and divided. Reconstruction was carried out by the modified Child method.

**Fig. 2:**
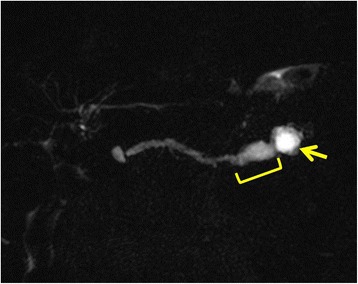
Magnetic resonance cholangiopancreatography. The size of the cyst in the pancreatic tail (*arrow*) and the diameter of the main pancreatic duct (MPD) gradually increased (*bracket*). The examination was performed 2 years and 6 months after the first surgery

**Fig. 3:**
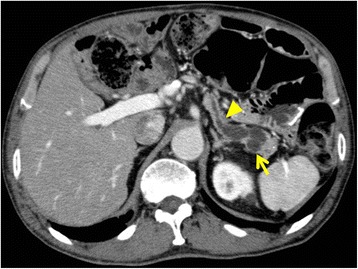
Computed tomography. A caliber change in the MPD at the pancreatic body was detected (*arrow head*). The cyst was located in the pancreatic tail (*arrow*)

**Fig. 4:**
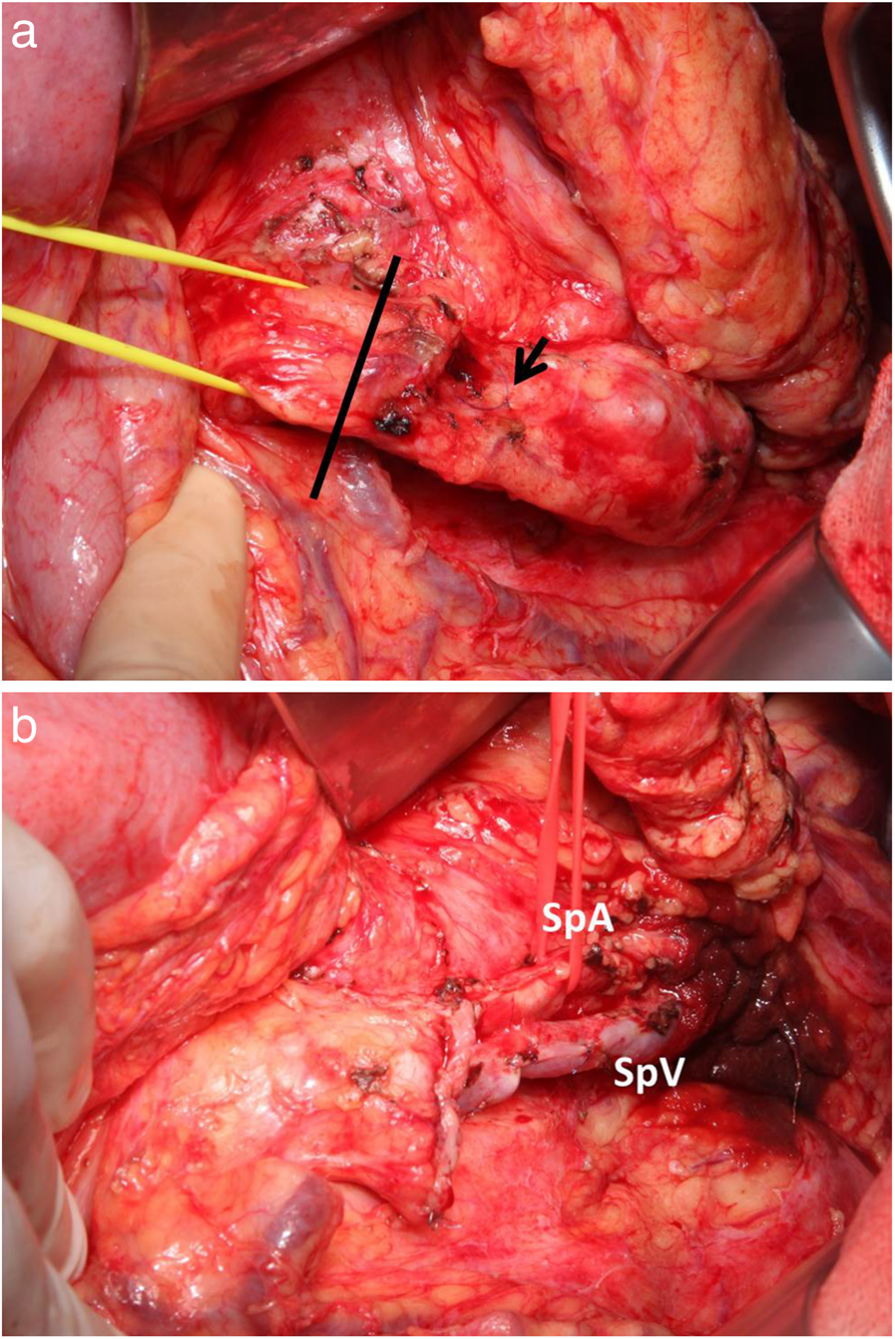
Operative findings. The pancreas was divided by a 2-cm margin from the point of caliber change of the MPD (*arrow*). The *line* indicated the resection line of the pancreas (**a**). The splenic artery and vein were preserved, and the remnant pancreas measured approximately 6 cm in length (**b**). *SpA* splenic artery, *SpV* splenic vein

A histopathological examination showed an intraductal papillary mucinous adenoma (IPMA) with mild-moderate atypia. Pancreatic epithelial cells in the stump showed no atypia. After surgery, the patient was followed up every 6 months. During the follow-up, the size of the cystic lesion in the pancreatic tail and the diameter of the MPD were gradually increasing. Two years and 6 months after surgery, CT and MRCP revealed proximal extension of the dilation of the MPD with a maximum diameter of 11 mm and a 16-mm-diameter cyst in the tail of the pancreas (Figs. 2 and 3). No intramural nodules in the MPD or cystic lesion were detected. The patient was diagnosed with IPMN with high-risk stigmata, and distal pancreatectomy (DP) with preservation of the spleen, namely MSPP, was performed. At laparotomy, the cystic lesion was located at the pancreatic tail and the proximal MPD was dilated. Intraoperative ultrasonography showed caliber change of the MPD at the pancreatic body. Dissection between the future remnant pancreas and splenic artery/vein was avoided in order not to injure the blood supply and drainage. The pancreas was divided with a 2-cm margin from that point (Fig. 4). The pancreatic resection margin was histologically negative. The length and volume of the remnant pancreas, as measured by postoperative CT, were approximately 6 cm and 10 ml, respectively. A histopathological examination showed an IPMA with mild-moderate atypia. No postoperative complications occurred, and the patient was discharged on postoperative day 8 smoothly.

**Fig. 5:**
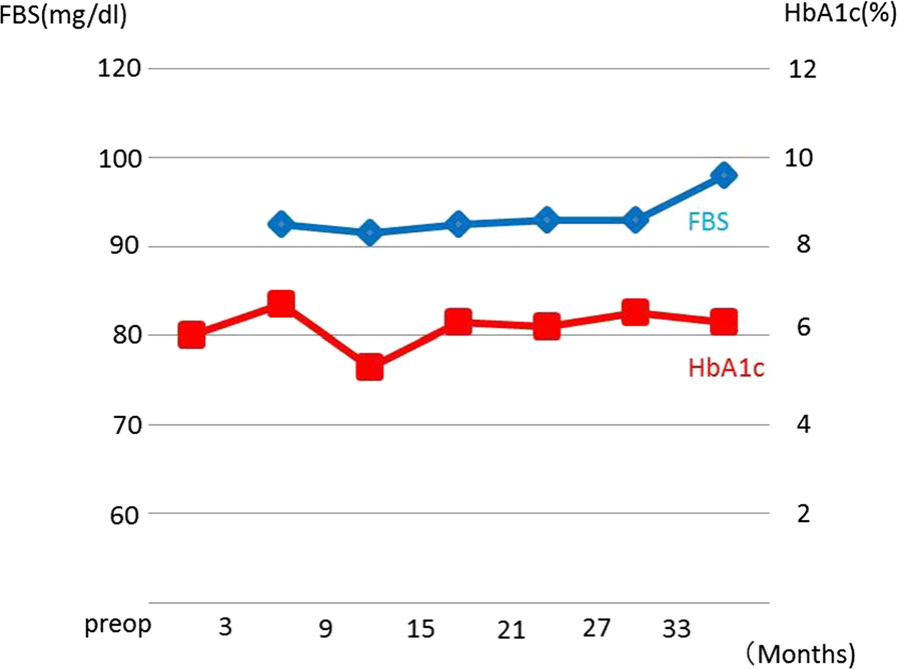
The changes in glucose tolerance after surgery

The patient had no diarrhea and weight loss without digestive enzymes. In the 3 years of follow-up since the last surgery, CT has never shown fatty liver or evidence of new lesions in the remnant pancreas. He also maintained favorable glucose tolerance without oral hypoglycemic agents or insulin (Fig. 5).

### Discussion

Siassi et al. [7] first reported the procedure of middle segment-preserving pancreatectomy (MSPP) after distal pancreatectomy in 1999. Since then, several case reports have been published regarding the safety and effectiveness of MSPP as an alternative to TP. Some authors have recommended MSPP for patients with multicentric noninvasive lesions, such as IPMNs, and pancreatic metastases, because lymphadenectomy was not necessary [6, 8–10]. Recently, as a minimally invasive surgery, laparoscopy-assisted MSPP was also reported [11]. Cheng et al. [6] reviewed 22 patients who underwent MSPP and showed that the overall morbidity was 40.9% (9/22), especially pancreatic fistula was 22.7% (5/22) with no mortality. Thus, MSPP was a safe and feasible procedure, and it has come to be widely performed for selected patients.

Postoperative exocrine and endocrine insufficiency is a major concern in pancreatectomy. Okano et al. [12] analyzed 227 patients who underwent PD or DP to determine the relationship between pancreatic volume and the postoperative pancreatic function. A volume of the remnant pancreas <24.1 ml was the only independent predictive factor for postoperative exocrine insufficiency; therefore, they recommended pancreatic enzyme therapy for these patients. Cheng et al. [6] reported that exocrine insufficiency after MSPP developed in 6 of 22 (27.3%) with a median follow-up of 18 months. Similarly, several other reports have described the relationship between the remnant volume of the pancreas and the postoperative endocrine function [13, 14]. Kang et al. [15] analyzed 101 patients who underwent DP and reported that a resected pancreatic volume >25% was a risk factor for endocrine impairment. Cheng et al. [6] also reported that endocrine insufficiency after MSPP developed in 7 of 22 patients (31.8%). Therefore, with respect to preserving the pancreas, these findings indicated that MSPP allows patients to avoid exocrine and endocrine insufficiency after surgery. In the present case, the volume of the remnant pancreas was very small (10 ml), which is 18.9% of the total pancreas; thus, the risk of postoperative exocrine and endocrine insufficiency was expected to be very high. Fortunately, our patient has maintained exocrine and endocrine functions without any kind of medication for more than 3 years after the second surgery. While the reason for this favorable outcome remains unclear, we speculate several reasons. First, at the initial surgery, the pancreatic function in the pancreatic body and tail was preserved because the pancreas head tumor was IPMN and not pancreatic cancer, and obstructive pancreatitis had not developed. Second, the resected pancreas in the last surgery was atrophic, and little of the functioning pancreas volume was lost. Third, we preserved the blood supply and drainage as much as possible. These conditions might have contributed to the good maintenance of the exocrine and endocrine functions of the remnant pancreas.

In addition, several reports have indicated that splenectomy is one of the risk factors for postoperative endocrine sufficiency. Hutchins et al. [16] analyzed 90 patients who underwent DP for chronic pancreatitis and reported that the incidence of DM after DP was lower in patients without splenectomy than in those with splenectomy (43 vs 72%). Tang et al. [17] retrospectively reviewed 82 patients who underwent DP with splenectomy and 78 patients who underwent DP without splenectomy for benign and borderline malignant tumors. They showed that fewer patients with splenectomy developed postoperative DM in comparison to patients who were treated without splenectomy (2.5 vs 12.2%). Thus, in cases in which an oncologically curative resection can be maintained (such as in the present case), the spleen should be preserved to provide a favorable endocrine function after surgery.

The preservation of the splenic artery was very important. First, although there have been no reports of pancreatic ischemia after MSPP, the blood supply to the remnant pancreas should be evaluated before surgery [8, 18]. The pancreatic body is mainly supplied by the splenic artery. In fact, in the present case, several small vessels to the pancreas, which originated from the splenic artery, were observed by preoperative CT. Second, the splenic artery has also been reported to be responsible for the main blood supply to the remnant stomach after distal gastrectomy [19]. In our case, the preservation of these vessels enabled the prevention of total gastrectomy.

## Conclusions

By performing DP with preservation of the spleen after PD and distal gastrectomy, namely MSPP for IPDA, we have preserved the postoperative exocrine and endocrine functions of the pancreas for more than 3 years since the last surgery. MSPP for selected patients therefore helps to ensure a high QOL after surgery.

## Abbreviations

BD-IPMN: Branch duct-type IPMN
CT: Computed tomography
DM: Diabetes mellitus
DP: Distal pancreatectomy
ERCP: Endoscopic retrograde cholangiopancreatography
EUS: Endoscopic ultrasonography
IPMA: Intraductal papillary mucinous adenoma
IPMN: Intraductal papillary mucinous neoplasm
MPD: Main pancreatic duct
MRCP: Magnetic resonance cholangiopancreatography
MSPP: Middle segment-preserving pancreatectomy
PD: Pancreatoduodenectomy
QOL: Quality of life
TP: Total pancreatectomy
